# The Importance of Perioperative Administration of an Anti-Hyperalgesic Drug in Burn Wounds

**DOI:** 10.5812/atr.7451

**Published:** 2012-10-14

**Authors:** Luca Dalle Carbonare, Micol Sandri, Vittorio Schweiger

**Affiliations:** 1 Department of Medicine, Clinic of Internal Medicine, University of Verona, Verona, Italy; 2 Intensive Care and Pain Therapy Centre, Department of Surgical Sciences, University of Verona, Verona, Italy

**Keywords:** Burns, Wounds, Pain

**Dear Editor,

We read with interest the article by Rimaz and coworkers about the beneficial effect of preoperative oral administration of a high single dose (1200 mg) of gabapentin on postoperative pain scores and morphine consumption in patients undergoing surgical debridement of burn wounds (1). The effect of preoperative administration of oral gabapentin in order to reduce postoperative pain scores and opioid consumption was analyzed in several studies. A systematic review of randomized controlled trials published by Ho and coworkers has shown that, in different types of surgery, the preoperative administration of a single dose of 1200 mg oral gabapentin produced a significant reduction in pain intensity at rest compared with placebo in the early and late postoperative period at 6 and 24 h after administration, respectively. In addition, some similar studies showed a significant reduction in opioid consumption and a delayed request for analgesia (2). As regard to the adverse effects, the administration of 1200 mg of oral gabapentin did not show significant differences with respect to placebo in a large part of published studies. Indeed, as highlighted by Turan and coworkers, the treatment group showed fewer episodes of vomiting and urinary retention, probably due to lower consumption of opioids in the postoperative period (3). However, administration of a single dose of 1200 mg of oral gabapentin in the preoperative period showed no serious adverse effects in the treated patients. The analysis of available data has revealed a number of limitations, in particular due to the different type of surgery. An interesting systematic review published by Mathiesen et al. focused on the use of gabapentin in the preoperative period in relation to the surgical procedures. Reported data showed that preoperative gabapentin administration in different dosages appears to be particularly effective in reducing postoperative opioid consumption particularly after abdominal hysterectomy and spinal surgery, while the use in other sort of surgeries revealed no significant results. In addition, a reduction in pain scores was demonstrated mostly in early postoperative period, probably due to higher gabapentin plasmatic level in the early postoperative phase (4). At a single preoperative oral dose of 1200 mg, gabapentin showed conflicting results in relation to different types of surgeries, with promising evidences only in abdominal hysterectomy, spinal surgery, radical mastectomy, thyroid and ear-nose throat surgery. However, these results should be confirmed by further investigations using the same methodology, in particular with respect to the type of anesthesia and rescue analgesia in the postoperative period. Currently, there are no other publications on the use of preoperative gabapentin in postoperative pain in burned patients. In this regard, we agree with the authors that the burn wounds, in relation to the particular type of damage, may benefit from perioperative administration of an anti-hyperalgesic drug. In fact, some nerve endings of skin nociceptors after burns are still intact or only partially injured and continue to generate pain impulses leading to primary hyperalgesia (5). However, further studies are needed to confirm these results, especially focused on the dose-response relationship of preoperative gabapentin, looking for the lowest effective dose. 

## References

[1] Rimaz S, Emir Alavi C, Sedighinejad A, Tolouie M, Kavosi S, Koochakinejad L (2012). Effect of Gabapentin on Morphine Consumption and Pain after Surgical Debridement of Burn Wounds: A Double-Blind Randomized Clinical Trial Study.. Arch Trauma Res..

[2] Ho KY, Gan TJ, Habib AS (2006). Gabapentin and postoperative pain--a systematic review of randomized controlled trials.. Pain..

[3] Turan A, Karamanlioglu B, Memis D, Hamamcioglu MK, Tukenmez B, Pamukcu Z (2004). Analgesic effects of gabapentin after spinal surgery.. Anesthesiology..

[4] Mathiesen O, Moiniche S, Dahl JB (2007). Gabapentin and postoperative pain: a qualitative and quantitative systematic review, with focus on procedure.. BMC Anesthesiol..

[5] Latarjet J, Choinere M (1995). Pain in burn patients.. Burns..

